# Anti‐obesity activity of *Heracleum moellendorffii* root extracts in 3T3‐L1 adipocytes

**DOI:** 10.1002/fsn3.2487

**Published:** 2021-07-21

**Authors:** Na Gyeong Geum, Ho Jun Son, Joo Ho Yeo, Ju Hyeong Yu, Min Yeong Choi, Jae Won Lee, Jueng Kyu Baek, Jin Boo Jeong

**Affiliations:** ^1^ Department of Medicinal Plant Resources Andong National University Andong Republic of Korea; ^2^ Forest Medicinal Resources Research Center National Institute of Forest Science Yeongju Republic of Korea; ^3^ Agricultural Corporation E·Farm Corp Yeongju Korea; ^4^ PINOGEN CO Ltd Andong Korea

**Keywords:** Adipocytes, adipogenesis, anti‐obesity activity, differentiation, *heracleum moellendorffii* root

## Abstract

It has been reported that *H. mollendorffii* roots (HMR) have various pharmacological activities such as anti‐inflammatory activity and immunostimulatory activity. However, the anti‐obesity activity of HMR has not been studied. Thus, we evaluated in vitro anti‐obesity of HMR in mouse preadipocytes, 3T3‐L1 cells. HMR reduced the lipid accumulation and triglyceride (TG) contents in 3T3‐L1 cells. HMR inhibited the protein expressions such as CCAAT/enhancer‐binding protein alpha (CEBPα), peroxisome proliferator‐activated receptor gamma (PPARγ), perilipin‐1, adiponectin, fatty acid‐binding protein 4 (FABP4), fatty acid synthase (FAS), and acetyl‐CoA carboxylase (ACC) related to the lipid accumulation of the mature adipocytes. In addition, HMR induced the proteasomal degradation of CEBPα related to the differentiation of the preadipocytes into the mature adipocytes by activating c‐Jun N‐terminal kinases (JNK) and glycogen synthase kinase 3 beta (GSK3β). Based on the results of this study, HMR inhibited the differentiation of preadipocytes into mature adipocytes through the CEBPα degradation via JNK and GSK3β activation and subsequently blocked lipid accumulation of mature adipocytes through inhibiting lipid accumulation‐related proteins such as CEBPα, PPARγ, perilipin‐1, adiponectin, FABP4, FAS, and ACC.

## Graphical abstract

Blurb For EtocHMR inhibited the accumulation of lipid and triglyceride in the mature adipocytes.

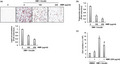

## 1 INTRODUCTION

Obesity is considered one of the most dangerous health problems worldwide today because it causes a variety of metabolic diseases such as diabetes, hypertension, fatty acid, cardiovascular disorders, and cancers (González‐Muniesa et al., 2017; Heymsfield & Wadden, 2017; Khandekar et al., 2011; Klil‐Drori et al., 2016). Obesity not only leads to metabolic diseases, but it has recently been reported that obesity increases the risk of death from COVID‐19 infection (Hussain et al., 2020).

Many people are making various efforts to treat obesity, such as diet control, exercise, lifestyle changes, prescription of anti‐obesity drugs, and surgery (Sun et al., 2016). Although the most ideal option of obesity treatment is known as a change in diet and lifestyle, it has been reported that obesity patients easily give up because it requires a long time (Sun et al., 2016; Thomas et al., 2014). Therefore, there is a need to provide an additional option to accelerate weight loss in obesity patients. Many people, including obesity patients, have used natural supplement products containing a variety of natural plants for weight loss. Thus, continuous search and development of new natural plants with anti‐obesity activity have been required.

*Heracleum moellendorffii* (*H. mollendorffii*), which grows naturally in Korea, China, and Japan, is an edible medicinal plant. Through many studies, it has been reported that *H. mollendorffii* leaves have various pharmacological activities such as detoxification and antioxidant activity and anti‐melanogenic activity (Alam et al., 2016; Bang et al., 2009; Park et al., 2010). In addition, *H. mollendorffii* roots have long been used as a traditional herbal medicine to treat inflammatory diseases such as arthritis, back pain, and fever (Alam et al., 2016). Recently, we reported that *H. mollendorffii* roots exert anti‐inflammatory activity and immunostimulatory activity (Kim et al., 2019; Son et al., 2020). However, there has been no study on the anti‐obesity activity of *H. mollendorffii* so far. Therefore, in this study, we evaluated the anti‐obesity activity of *H. mollendorffii* roots through the inhibition of lipid accumulation in 3T3‐L1 cells.

## 2 MATERIALS AND METHODS

### 2.1 Chemical reagents

Kinase inhibitors such as extracellular signal‐regulated kinases 1/2 (ERK1/2) inhibitor PD98059, p38 inhibitor SB203580, c‐Jun N‐terminal kinases (JNK) inhibitor SP600125, IκB kinase (IκK) inhibitor BAY 11–7082, glycogen synthase kinase 3 beta (GSK3β) inhibitor LiCl, and Oil Red O staining solution for lipid droplet staining were purchased from Sigma‐Aldrich (St. Louis, MO, USA). The primary and secondary antibodies used for Western blot analysis were purchased from Cell Signaling (Bervely).

### 2.2 HMR preparation

*H. moellendorffii* roots (HMR) were prepared according to the protocol of our previous study (Son et al., 2020). HMR was dissolved in sterile distilled water, aliquoted in small portions, and stored at −80ºC until use.

### 2.3 Cell culture

3T3‐L1 cells, a mouse preadipocytes, were purchased from American Type Culture Collection (Manassas). 3T3‐L1 cells were cultured in Dulbecco's Modified Eagle medium (DMEM)/F‐12 1:1 Modified medium (Lonza) containing 10% bovine calf serum, 100 U/ml penicillin and 100 μg/ml streptomycin at 37ºC under a humidified atmosphere of 5% CO_2_. Two days after the cells reached confluence (designated as D0), 3T3‐L1 cells were incubated with DMI media (DMEM/F‐12 containing 10% fetal bovine serum (FBS), 1 μM dexamethasone, .5 mM 3‐isobutyl‐1‐methylxanthine, and 10 μg/ml insulin) for 48 hr (D0‐D2). Then, on day 2, 3T3‐L1 cells were cultured in DMEM/F‐12 containing 10% FBS and 10 μg/ml insulin for 48 hr (D2‐D4). After that, the medium (DMEM/F‐12 containing 10% FBS) was then changed every 2 days (D4 and D6). The treated cells were recovered on days 8 (D8).

### 2.4 Oil Red O staining

After the treatment was completed, 3T3‐L1 cells were gently washed three times with 1 X phosphate‐buffered saline (PBS) and then fixed with 10% formalin for 1 hr at the room temperature. After fixation, 3T3‐L1 cells were washed three times with distilled water and then 3T3‐L1 cells was left with 60% isopropanol for 5 min at the room temperature. After 5 min, 3T3‐L1 cells were completely dried and stained with Oil Red O solution (60% isopropanol and 40% water) for 20 min at the room temperature. After washing with distilled water five times, stained lipid droplets of 3T3‐L1 cells were visualized and photographed with a light microscope (Olympus) and then dissolved in 100% isopropanol and quantified by measuring the absorbance at 500 nm with a microplate reader (Human Cop., Xma‐3000PC).

### 2.5 Measurement of intracellular triglycerides (TG)

After the treatment was completed, 3T3‐L1 cells were washed with cold 1 X PBS and collected into the sonication buffer (25 mM Tris‐buffer containing 1 mM EDTA, pH 7.4) using a cell scraper and homogenized by sonication for 5 min. The cell lysate was centrifuged at 8,000 g for 10 min at 4ºC and the cellular protein was determined by bicinchoninic acid protein (BCA) protein assay (Thermo Fisher Scientific). Intracellular TG quantification was measured using Triglyceride Quantification Kit (BioVision, Milpitas) according to the manufacturer's protocol.

### 2.6 Cell proliferation assay

After the treatment was completed, the number of 3T3‐L1 cells was measured using NucleoCounter NC‐250 (Chemometec) according to the manufacturer's protocol.

### 2.7 Western blot analysis

Western blot analysis was performed according to the protocol of our previous study (Son et al., 2020). The samples for SDS‐polyacrylamide gel electrophoresis (SDS‐PAGE) were prepared by extracting proteins from 3T3‐L1 cells with radio‐immunoprecipitation assay buffer (Boston Bio Products) containing protease inhibitor (Sigma‐Aldrich) and phosphatase inhibitor (Sigma‐Aldrich) and quantifying the protein amounts by BCA assay (Thermo Fisher Scientific). After sample preparation, the protein (25 μg/well) was separated by SDS‐PAGE, and then proteins separated on the gel were transferred to the polyvinylidene fluoride (PVDF) membrane. After the transfer, the PVDF membrane was blocked with 5% nonfat milk in Tris‐buffered saline containing .05% Tween 20 (TBS‐T) at room temperature for 1 hr, and then, the PVDF membranes were treated with the primary antibodies in 5% bovine serum albumin in TBS‐T at 4ºC for overnight. After the primary antibody treatment was completed, the PVDF membranes were treated with the secondary antibodies in 5% nonfat milk in TBS‐T at room temperature for 1 hr. Chemiluminescence was detected with ECL Western blotting substrate (Amersham Biosciences) and visualized using LI‐COR C‐DiGit Blot Scanner (Li‐COR Biosciences). The density of Western blot bands was calculated using the software UN‐SCAN‐IT gel version 5.1 (Silk Scientific Inc.).

### 2.8 Reverse transcription polymerase chain reaction (RT‐PCR)

RT‐PCR was performed according to the protocol of our previous study (Son et al., 2020). The isolation of total RNA from 3T3‐L1 cells and the synthesis of cDNA using the isolated total RNA were carried out using a RNeasy Mini Kit (Valencia) and a Verso cDNA Kit (Thermo Scientific), respectively. The amplification of the target gene was performed using a PCR Master Mix Kit (Promega) and the primers. The sequences of the primers used in this study were as follows: PPARγ: forward 5’‐gaaagacaacggacaaatcacc‐3’ and reverse 5’‐gggggtgatatgtttgaacttg‐3’, CEBPα: forward 5’‐ttacaacaggccaggtttcc‐3’ and reverse 5’‐aactccagtccctctgggat‐3’, and GAPDH: forward 5’‐ggactgtggtcatgagcccttcca‐3’ and reverse 5’‐actcacggcaaattcaacggcac‐3’. The PCR results were visualized using agarose gel electrophoresis. The density of bands for PCR products (DNA) was calculated using the software UN‐SCAN‐IT gel version 5.1 (Silk Scientific Inc).

### 2.9 Statistical analysis

All the data are shown as mean ± *SD* (standard deviation). Statistical significance was determined by Student's *t* test. Differences with *P or ^#^ *p* < .05 were considered statistically significant.

## 3 RESULTS AND DISCUSSION

### 3.1 HMR suppresses excessive lipid accumulation in 3T3‐L1 cells

Long‐term imbalanced diets cause obesity, which results in inducing various metabolic diseases such as diabetes, hypertension, cardiovascular disease, and cancer (Gammone & D’Orazio, 2015). Therefore, the most effective strategy for preventing these metabolic diseases has been considered to be the prevention of obesity (Gammone & D’Orazio, 2015).

Adipocytes are known to play an important role in energy homeostasis and lipid metabolism, but an increase in the number and size of adipocytes in adipose tissue induces obesity development (Hwang et al., 2017). Thus, inhibiting lipid accumulation in adipocyte has been considered an important target in preventing or treating obesity (Wang et al., 2020).

**FIGURE 1:**
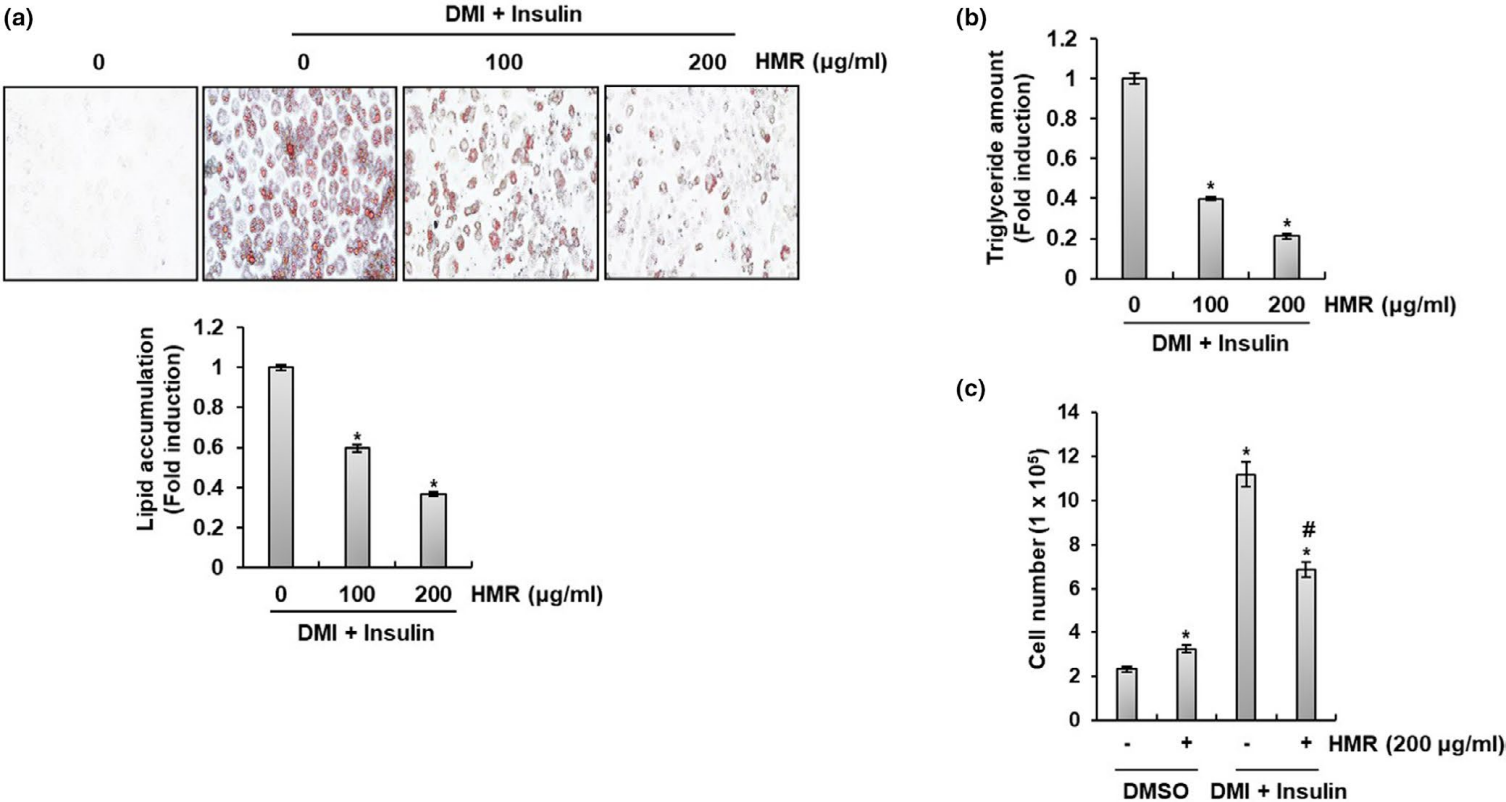
Effect of HMR on the lipid accumulation and TG content in 3T3‐L1 cells. (A) Lipid accumulation was determined by measuring Oil Red O staining. (B) TG content was determined by Triglyceride Quantification Kit. (C) Cell number was determined by NucleoCounter NC‐250. *P or #*p* < .05 compared to the cells without HMR treatment

To evaluate whether HMR inhibits excessive lipid accumulation in adipocyte, lipid accumulation and triglyceride (TG) content in 3T3‐L1 cells treated with HMR were measured. First, lipid accumulation in 3T3‐L1 cells was measured by Oil Red O staining and, as a result, it was observed that lipid accumulation in HMR‐treated 3T3‐L1 cells was decreased compared to cells not treated with HMR (Figure 1a). In addition, HMR reduced TG content of 3T3‐L1 cells in a concentration‐dependent manner (Figure 1b). We also investigated the effect of HMR on the growth of preadipocytes and adipocytes. As shown in Figure 1c, HMR slightly increased preadipocytes proliferation, while the proliferation of adipocytes was inhibited by HMR treatment. Taken together, it is thought that HMR inhibits excessive lipid accumulation in adipocytes.

### 3.2 HMR suppresses the protein expression associated with lipid accumulation in 3T3‐L1 cells

**FIGURE 2:**
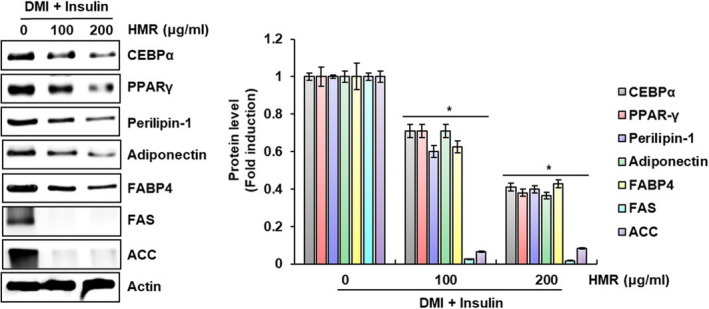
Effect of HMR on the expression of protein related to the lipid accumulation in 3T3‐L1 cells. The protein levels were determined by Western blot analysis. Actin was used as a loading control. *p < .05 compared to the cells without HMR

It has been reported that various factors, including CEBPα, PPARγ, perilipin‐1, adiponectin, FABP4, FAS, and ACC, play an important in lipid accumulation of adipocytes after differentiated from preadipocytes (Lee et al., 2010). CEBPα and PPARγ known as transcription factors highly expressed in adipose tissue are known to increase lipid accumulation by directly regulating FAS and ACC related to adipogenesis (Schultz et al., 2000; Joseph et al., 2002). Perilipin‐1 has been reported to promote lipid droplet formation in adipocytes and block lipolysis of adipocytes. Thus, perilipin‐1 is considered a new target for the treatment of obesity (Noureldein, 2014). In addition, it has been reported that adiponectin, an adipokine hormone secreted from adipose tissue, promotes the differentiation of adipocytes and lipid accumulation (Fu et al., 2005) and FABP4 regulates intracellular lipid accumulation (Scifres et al., 2011). Thus, to evaluate whether the inhibitory activity of HMR against lipid accumulation in 3T3‐L1 cells is due to inhibiting the expression of lipid accumulation‐related factors, we investigated the effect of HMR on the protein levels such as CEBPα, PPARγ, perilipin‐1, adiponectin, FABP4, FAS, and ACC in HMR‐treated 3T3‐L1 cells. As shown in Figure 2, the levels of protein expression such as CEBPα, PPARγ, perilipin‐1, adiponectin, FABP4, FAS, and ACC remarkably were reduced in 3T3‐L1 cells treated with HMR. From these results, it is thought that HMR inhibits lipid accumulation by blocking the expression of lipid accumulation‐related proteins in adipocytes.

### 3.3 HMR suppresses early and late adipogenesis in 3T3‐L1 cells

Adipogenesis, the process of differentiation of preadipocytes into mature adipocytes with lipid accumulation, is closely related to the development of obesity (Ando et al., 2019). It is known that the adipogenesis process is divided into an early stage from day 0 to day 2 and a late stage from day 3 to day 8 (Inokawa et al., 2016; Tomiyama et al., 1999).

**FIGURE 3:**
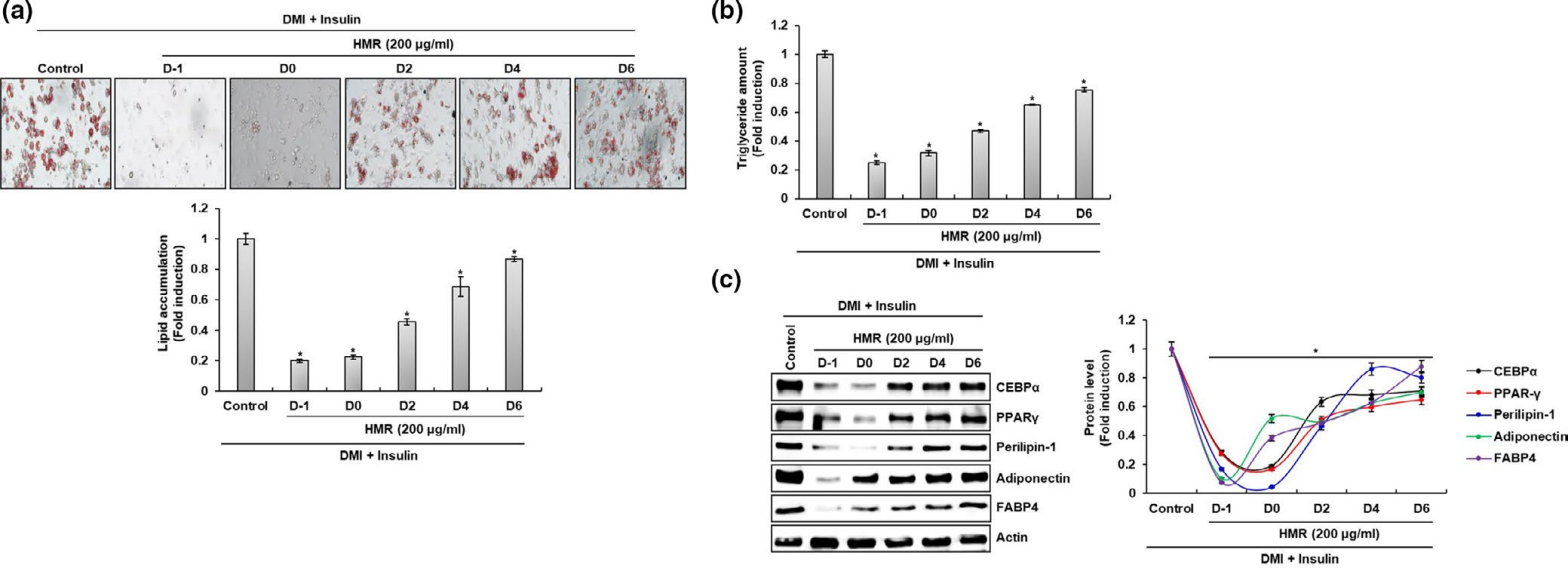
Effect of HMR on the early and late adipogenesis in 3T3‐L1 cells. All 3T3‐L1 cells were incubated with DMI from D0 to D2. Then, on D2, 3T3‐L1 cells were treated with 10 μg/ml of insulin from D2 to D4. After that, the medium (DMEM/F‐12 containing 10% FBS) was then changed every 2 days (D4 and D6). HMR (200 μg/ml) was treated one day before DMI was treated (HMR D‐1), treated concurrently with DMI (HMR D0), treated concurrently with insulin (HMR D2), treated when the medium was changed every 2 days (HMR D4 and HMR D6). The treated cells were recovered on D8. (A) Lipid accumulation was determined by measuring Oil Red O staining. (B) TG content was determined by Triglyceride Quantification Kit. (C) The protein levels were determined by Western blot analysis. Actin was used as a loading control. *p < .05 compared to the cells without HMR treatment

To evaluate the effect of HMR on early and late phase in the adipogenesis of 3T3‐L1 cells, lipid accumulation by Oil Red O staining, TG content, and protein expression by Western blot analysis were investigated after HMR was treated to 3T3‐L1 cells by time. As shown in Figure 3a, 3b, HMR treatment at D‐1 and D0 (early phase) completely reduced lipid accumulation and TG content in 3T3‐L1 cells. In addition, lipid accumulation and TG content decreased remarkably in 3T3‐L1 cells treated with HMR at D2, D4, and D6 (late phase). Also, HMR inhibited protein expression such as CEBPα, PPARγ, Perilipin‐1, Adiponectin, and FABP4 at the early and late phase of the adipogenesis in 3T3‐L1 cells (Figure 3c). Considering these results, HMR is thought to inhibit not only the early stages of adipogenesis but also the late stages.

### 3.4 HMR induces proteasomal degradation of CEBPα involved in the differentiation of 3T3‐L1 cells

**FIGURE 4:**
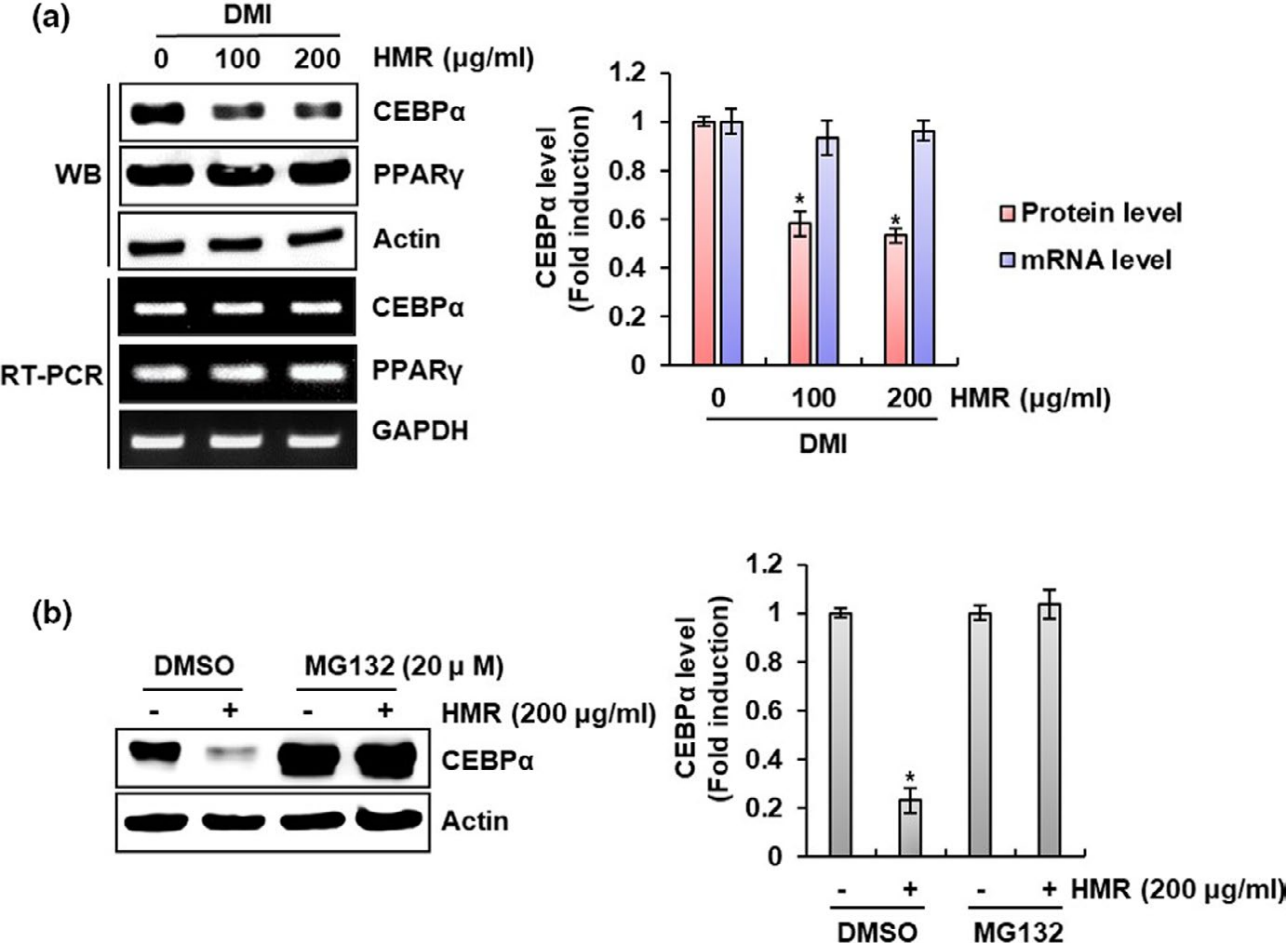
Effect of HMR on the expression of CEBPα and PPARγ related to the differentiation of preadipocytes into the mature adipocytes in 3T3‐L1 cells. (A) 3T3‐L1 cells were treated with HMR in presence of DMI for 48 hr. The protein levels were determined by Western blot analysis and RT‐PCR analysis. Actin and GAPDH were used as a loading control for Western blot analysis and RT‐PCR analysis, respectively. (B) 3T3‐L1 cells were treated with DMI for 24 hr and then co‐treated with HMR and MG132 for 24 hr. The protein levels were determined by Western blot analysis. Actin was used as a loading control. *p < .05 compared to the cells without HMR treatment

CEBPα and PPARγ are known as transcription factors involved in not only lipid accumulation in mature adipocytes but also differentiation from preadipocytes into mature adipocytes (Yang et al., 2018). To evaluate whether HMR inhibits differentiation from preadipocytes to mature adipocytes, the expression levels of CEBPα and PPARγ were investigated in HMR‐treated 3T3‐L1 cells. As shown in Figure 4a, HMR attenuated CEBPα protein level but not mRNA level in HMR‐treated 3T3‐L1 cells. However, the level change of PPARγ did not occur at the protein and mRNA level in 3T3‐L1 cells. These results suggest that decrease in CEBPα protein by HMR is due to the induction of CEBPα proteasomal degradation by HMR. Indeed, it was confirmed that the CEBPα degradation in adipocytes inhibits the differentiation of preadipocytes into mature adipocytes (Pal et al., 2013). Thus, to verify whether HMR induces the degradation of CEBPα protein, the level of CEBPα protein was investigated in the cells treated with HMR and MG132 (proteasome inhibitor). As a result, CEBPα protein level was decreased by HMR in 3T3‐L1 cells not treated with MG132, but HMR‐induced reduction of CEBPα protein level did not occur in MG132‐treated 3T3‐L1 cells (Figure 4b). In view of these results, it is thought that HMR inhibits the differentiation of preadipocytes into mature adipocytes by degrading CEBPα protein.

### 3.5 HMR‐induced degradation of CEBPα protein is dependent on JNK and GSK3β activation

**FIGURE 5:**
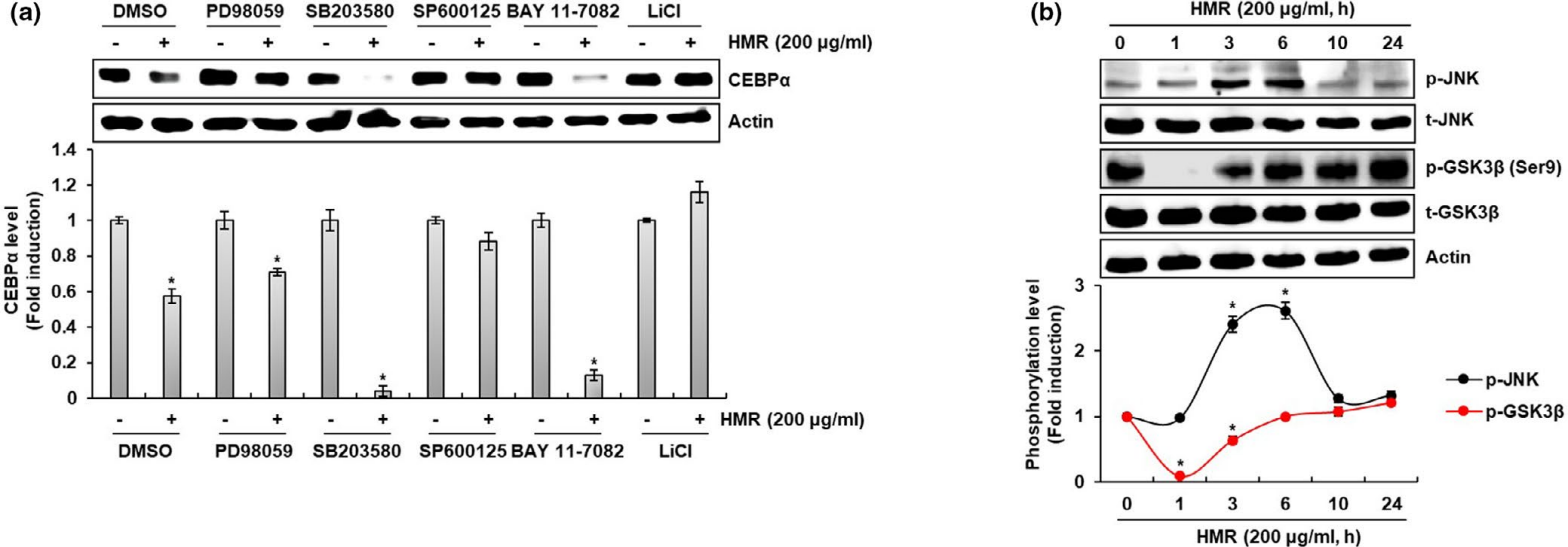
Effect of the upstream kinases on HMR‐mediated degradation of CEBPα in 3T3‐L1 cells. (A) 3T3‐L1 cells were treated with DMI for 48 hr and then co‐treated with HMR, PD98059 (20 μM), SB203580 (20 μM), SP600125 (20 μM), BAY 11–7082 (20 μM), and LiCl (20 mM) for 24 hr. (B) 3T3‐L1 cells were treated with DMI for 48 hr and then co‐treated with HMR for the indicated times. The protein levels were determined by Western blot analysis. Actin was used as a loading control. *p < .05 compared to the cells without HMR treatment

Proteasomal degradation of the intracellular protein is regulated by various upstream kinases. To investigate the upstream kinases involved in HMR‐induced degradation of CEBPα protein, 3T3‐L1 cells were pretreated with PD98059 (ERK1/2 inhibitor), SB203580 (p38 inhibitor), SP600125 (JNK inhibitor), BAY 11–7082 (IκK inhibitor), or LiCl (GSK3β inhibitor) and then co‐treated with HMR. As shown in Figure 5a, HMR reduced the level of CEBPα protein in presence of PD98059, SB203580, and BAY 11–7082. However, the inhibition of JNK by SP600125 and GSK3β by LiCl blocked HMR‐mediated reduction of CEBPα protein level. Thus, we confirmed that HMR induces the activation of JNK and GSK3β. As a result, HMR increased phosphorylation, an active form of JNK, and decreased phosphorylation, an inactive form of GSK3β (Figure 5b). Taken together, this result suggests that HMR causes CEBPα proteasomal degradation by activating JNK and GSK3β.

## 4 CONCLUSION

In summarizing the overall results derived from this study, HMR inhibits the differentiation of preadipocytes into mature adipocytes through the CEBPα degradation via JNK and GSK3β activation, and subsequently blocking lipid accumulation of mature adipocytes through reducing the level of the adipogenesis‐related proteins such as CEBPα, PPARγ, perilipin‐1, adiponectin, FABP4, FAS, and ACC. These results can provide evidence for the anti‐obesity activity of HMR, and HMR can be used as an important natural product for the prevention and treatment of the obesity.

## CONFLICT OF INTEREST

The authors declare no conflict of interest.

## AUTHOR CONTRIBUTIONS

**Na Gyeong Geum:** Data curation (lead); Investigation (lead); Writing‐original draft (supporting). **Ho Jun Son:** Data curation (supporting); Investigation (supporting); Writing‐original draft (supporting). **Joo Ho Yeo:** Data curation (supporting); Investigation (supporting); Writing‐original draft (supporting). **Ju Hyeong Yu:** Formal analysis (supporting); Investigation (supporting); Writing‐review & editing (supporting). **Min Yeong Choi:** Formal analysis (supporting); Investigation (supporting); Writing‐review & editing (supporting). **Jae Won Lee:** Formal analysis (supporting); Supervision (supporting); Writing‐original draft (supporting); Writing‐review & editing (supporting). **Jueng Kyu Baek:** Methodology (supporting); Writing‐original draft (supporting); Writing‐review & editing (supporting). **Jin Boo Jeong:** Conceptualization (lead); Funding acquisition (lead); Methodology (lead); Project administration (lead); Supervision (lead); Writing‐review & editing (lead).
